# The risk of cancer in patients with primary Sj**ö**gren syndrome: a single-center study from Turkey

**DOI:** 10.55730/1300-0144.5350

**Published:** 2021-11-13

**Authors:** Bengisu ASLAN, Tahir Saygın ÖĞÜT, Funda ERBASAN, Melis DİLBİL, Ece ÇELİK, Mustafa Ender TERZİOĞLU, Veli YAZISIZ

**Affiliations:** 1 Division of Rheumatology, Department of Internal Medicine, Akdeniz University, Antalya, Turkey; 2 Division of Allergy-Immunology, Department of Internal Medicine, Akdeniz University School of Medicine, Antalya, Turkey

**Keywords:** Sjögren syndrome, cancer, lymphoma, incidence, malignancy

## Abstract

**Background:**

The aim of this study is to determine the risk of cancer in patients with primary Sjögren syndrome (pSS) from a single center in Turkey.

**Methods:**

Clinical data of the subjects with pSS were retrospectively analyzed. The incidence of cancer for general population was obtained from GLOBOCAN 2018. Age- and sex-specific standardized incidence ratios (SIR) of solid and hematological cancers were calculated compared with the general population.

**Results:**

Four hundred thirty patients with pSS were included in the study. The majority of the patients were female (n = 396, 92.1%), and the mean age was 58.6 ± 12.0 years. Thirty-four patients (7.9 %) were diagnosed with cancer (26 solid and 8 hematological) during follow-up. The SIR for all cancers was 2.45 (95% CI, 1.625–3.275). The SIR was 2.42 (95% CI, 1.542–3.298) for solid cancers and 8.42 (95% CI, 2.394 – 14.446) for hematological cancers. The most diagnosed malignancies were breast cancer (n = 6), ovarian cancer (n = 6), and non-Hodgkin lymphoma (NHL) (n = 4). There was an increased risk for ovarian cancer (SIR 12.76, 95% CI, 2.545–22.975). The SIR values were 2.08 (95% CI, 0.419–3.741) and 10.81 (95% CI, 0.216–21.404) for breast cancer and NHL, respectively.

**Conclusion:**

The risk of hematological and solid cancers was higher in the patients with pSS when compared to general population. In our pSS cohort, the risk for ovarian cancer was found to be increased, which has not been previously reported in the literature.

## 1. Introduction

Primary Sjögren syndrome (pSS) is a systemic autoimmune disease that mainly affects secretory glands [1]. Most patients with pSS suffer from dryness of mouth and eyes. Some patients have systemic diseases involving skin, joints, lungs, nervous system, muscles, and hematopoietic system [2–4]. Primary SS is characterized by lymphocytic infiltration of affected tissues. Lymphoid aggregates and ectopic lymphoid structures containing T and B lymphocytes develop in response to chronic inflammation in nonlymphoid organs. Antigen-driven T cell-mediated B cell hyperreactivity, leading to autoreactive B cell activation, plays an important role in the pathogenesis of pSS [5, 6].

Clinical studies to clarify whether the survival of the patients with pSS is similar to the general population are in progress. The prognosis varies depending on disease severity, extent, and comorbidities. Systemic organ involvements and malignancies, including lymphoma, may contribute to an increase in mortality rates. Our clinical data revealed that the mortality rate of the patients with pSS was higher than that of the general population, the patients with interstitial lung disease had lower survival rates, and malignancy may also be associated with a worse prognosis [7].

It is known that the risk of lymphoma and solid organ cancers is increased in patients with pSS [8–10]. Many clinical studies showing increased non-Hodgkin lymphoma (NHL) prevalence in pSS have already been published [11–13]. In a metaanalysis, it was found that the frequency of developing lymphoma at 5, 15, 20 years after the diagnosis were 4%, 10%, and 18%, respectively [14]. The rate of malignancy among patients with pSS during an 18-year follow-up in Sweden was 11.5% [15]. On the other hand, malignancy was diagnosed in 2.2% of a Chinese pSS cohort during 15-year follow-up [8]. Primary SS is also associated with the development of solid cancers. In the literature, there are clinical studies reporting enhanced risk for the development of thyroid, oral cavity, and stomach cancers, while a lower risk for colon and breast cancers [16, 17, 18].

The frequency of malignant diseases may be different among populations depending on the genetic and environmental factors [19]. The development of cancers is affected by the differences in sex, age, ethnicity, diet and environmental exposures, and also chronic diseases. The predictive factors for the development of lymphoma in pSS are the presence of glandular enlargement, cutaneous vasculitis, glomerulonephritis, cryoglobulinemia, lymphadenopathy, neutropenia, splenomegaly, and hypocomplementemia [20–22]. The risk factors and predictors for the development of solid cancers in pSS are not clear as in hematologic malignancies. In addition, there is no published report concerning the frequency and risk factors for malignancy in Turkish patients with pSS. The aim of this study was to determine the risk of cancer in a Turkish pSS cohort from a single-center.

## 2. Materials and methods

### 2.1. Study population

Patients who were diagnosed with pSS according to American College of Rheumatology 2012 criteria at Akdeniz University Hospital between January 2004 and December 2018 were included in the study [23]. Patients who had other rheumatic diseases, in addition to pSS, were excluded. The clinical characteristics and laboratory findings of the patients were retrospectively collected from their medical records and the hospital’s electronic database.

Histopathologically detected cancers were determined and classified according to the International Classification of Diseases (ICD 10) criteria, and cancers developed after the diagnosis of pSS were included in the analysis.

### 2.2. Patient variables

Clinical variables such as age, sex, disease duration, and follow-up period of the patients were analyzed. In addition, the presence of dry eyes or dry mouth, joint symptoms/findings, Raynaud phenomenon, and lung involvement were recorded. Laboratory findings including complete blood counts, biochemical tests, erythrocyte sedimentation rate (ESR), C-reactive protein (CRP), antinuclear antibodies (ANA), rheumatoid factor (RF), anti-ro and anti-La antibodies, complement 3 (C3), complement 4 (C4), and immunoglobulin levels were recorded after review of electronic medical records. Normal laboratory values were defined according to our laboratory reference values. Anemia, leukopenia, and thrombocytopenia were defined as hemoglobin level less than 12 mg/dL, a leukocyte count less than 4.0×10^9^/L, and a thrombocyte count less than 100×10^9^/L. If ESR and CRP values were over 20 mm/h and 0.5 mg/dL, respectively, they were considered as high. The cut-off value for ANA positivity was considered as > 1/100 titer. The presence of anti-Ro/anti-La antibodies was determined by extractable nuclear antigen panel, and RF positivity was determined by nephelometric test method. Cut-off levels for C3 and C4 were 80 mg/dL and 10 mg/dL, respectively.

This study was approved by the medical ethics committee of Akdeniz University Hospital and complied with the principles of the Declaration of Helsinki.

### 2.3. Statistical analysis

Statistical analyses were performed using SPSS software, version 13.0 (SPSS, Chicago, IL, USA). The data were expressed as means ± standard deviation (SD) for continuous variables. Continuous variables were compared using the Student’s t-test. Categorical variables were compared using a chi-square test. Logistic regression analyses were used to determine the risk factors associated with the development of malignancy. A value of p < 0.05 was considered significant.

The person-years of follow-up for patients with pSS were calculated from the date of diagnosis to the last visit or cancer diagnosis (whichever occurred earliest). Cancer data of the Turkish population were determined from GLOBOCAN 2018 [24]. The standardized incidence ratios (SIR), also stratified by sex and age, were computed as the ratio of observed to expected cancers in the patients with pSS. The 95% confidence intervals (CI) of the SIR were also calculated. The incidence of cancer for the Turkish population was obtained from GLOBOCAN 2018. There were no patients diagnosed with pSS under 20 years old in this study group, so, age- (over 20 years old) and gender-specific standardized incidence ratios (SIR) were calculated. The incidence of cancer for the Turkish population older than 20 years was reported to be 363/100.000 (460/100.000 for males, 292/100.000 for females) in GLOBOCAN 2018 Turkey database.

## 3. Results

### 3.1. Characteristics of the subjects

There were 430 patients with pSS who were older than 18 years and diagnosed between the years 2004–2018 (Table 1). The mean age of the patients was 58.6 ± 12.0 years (Min-Max: 21–80 years). The majority of patients were female (n = 396, 92.1%). During follow-up period, 34 patients (7.9%) were diagnosed with malignant disease, of which 26 were solid tumors, and 8 were hematological cancers. The most frequent types of cancers were breast (1.4%) and ovarian (1.4%) cancers. All hematological cancers were seen in female patients. The results demonstrated the prevalence of malignancy was higher in male than female patients (11.8% versus 7.6%). But it was not statistically significant (p: 0.330). The types of cancers detected and their distribution among patients are shown in detail in Table 2.

The clinical characteristics and laboratory data of the patients with and without cancer were compared (Table 1). The patients with cancer were older (66.2 ±10.2 vs. 57.9±12.0 years, p < 0.001) and their disease duration was longer (9.30±5.2 vs. 7.25±4.6 years, p = 0.003) than the subjects with no cancer. The incidence of thrombocytopenia and increased CRP levels detected at the time of diagnosis were significantly higher in the patients with cancer when compared to the subjects with no cancer (18.2% vs. 3.8%, p < 0.001, and 56% vs. 34.1%, p = 0.027, respectively).

### 3.2. Standardized incidence ratios (SIRs) for cancer

The rates of cancer incidence for this cohort and Turkish population in general, both expressed as 100,000 patient-years, were given in Table 3. The SIR was calculated by using the observed and expected number of patients, and it’s 95% CI. The SIR (95% CI) could not be calculated for cancer subtypes less than four. Therefore, the SIRs were given for all cancers, solid/hematological, and the most commonly seen types (breast, ovarian, and non-Hodgkin lymphomas).

Compared with the general population, the SIR was 2.45 (95% CI 1.625–3.275) for all cancers and was higher in men than in women (2.96 vs. 2.91) (Table 3). The SIR was 2.42 (95% CI 1.542–3.298) for solid cancers and 8.42 (95% CI 2.394–14.446) for hematological cancers. Concerning the types of cancer, there was an increased risk for ovarian cancers (SIR 12.76; 95% CI 2.545–22.975). The SIRs were 2.08 (95% CI 0.419–3.741) and 10.81 (95% CI 0.216–21.404) for breast cancer and NHL, respectively.

The logistic regression analysis indicated that older age (p = 0.005), presence of leukopenia (p = 0.30) and thrombocytopenia (p < 0.01), and use of corticosteroids (p < 0.01) were independent risk factors of developing malignancies in pSS patients (Table 4).

## 4. Discussion

This study was conducted to determine the risk of cancer in patients with pSS. The results revealed that the overall cancer incidence rates, both for solid and hematologic cancers, were higher in the patients with pSS when compared to age and gender-matched populations. There was a 2.45-fold overall increased risk for cancer (SIR: 2.45, 95% CI 1.625–3.275). This study is the first report on the risk of cancer and cancer types detected in Turkish patients with pSS.

Although it is already known that the incidence of cancers, especially lymphomas, is increased in the patients with pSS, the exact rates are unknown. Igoe et al. reported that the patients with pSS had a greater risk for developing lymphoma (SIR, 10.5–44), multiple myeloma (SIR, 3.3–1.5), and lung cancer (SIR,1.29–4.5) when compared to healthy individuals [25]. The risk of NHL in pSS was higher than that reported for patients with systemic lupus erythematosus (SLE) and rheumatoid arthritis (RA) [25]. In a metaanalysis, Liang Y et al. reported an overall increased risk for cancers, NHL, and thyroid cancer in pSS, and stated that it has not yet been known whether the increased risk of overall malignancy was due to relatively high prevalence of NHL [9]. Some authors have suggested the increased overall cancer risk was associated with hematological cancers [26]. There is a debate as to whether the risk of developing solid cancers is increased in pSS. In addition, an increase in the incidence of nonhematological malignancies has not been proven in some studies [15, 22]. Our findings confirmed an increased risk of solid cancers in patients with pSS. The SIR was calculated as 2.42 (95% CI 1.542–3.298) for solid cancers detected in our pSS cohort. Our findings were consistent with the results of recently published studies that revealed a high risk for solid cancers [10,27].

Lymphocytic infiltration in target tissues and chronic lymphocyte activation are key features of pSS. B cell-activating factor (BAFF), cytokines, activation of Nf-kB, and germinal center-like (GC-like) structures are important for the development of lymphoma in pSS [28]. Our results show that Turkish patients with pSS have lower risk for developing NHL (SIR: 10.81, 95% CI 0.216–21.404) when compared to Chinese (SIR:48.1, 95% CI 20.7–94.8), Argentine (SIR:41.40, 95% CI 10.12–102.1) Swedish (SIR:15.57, 95% CI 7.8–27.9), and British (SIR:37.5, 95% CI 20.7–67.6) patients [8,10,15,22], while it washigher than Spanish (SIR:6.04, 95% CI 3.43–10.64), Korean (SIR:6.42, 95% CI 4.09–8.76), and Finn (SIR:8.7, 95% CI 4.3–15.5) patients with pSS [16,27,29]. The main reason for not detecting statistical difference for NHL in this study is the small number of patients diagnosed with NHL.

The current evidence about the development of NHL in pSS is increasingly growing. The clinical and laboratory predictors, such as the presence of salivary gland enlargement, lymphadenopathy, Raynaud phenomenon, anti-Ro/SSA or/and anti-La/SSB autoantibodies, RF positivity, monoclonal gammopathy, and hypocomplementemia were identified to be independent predictors for the development of NHL [30]. The reason why the risk is different for different populations with pSS is another issue that needs to be investigated. It can be explained by international, multicenter registries covering a large number of cases. A multinational study showed that the systemic phenotype of pSS was influenced by geoepidemiological players and personal determinants such as age, gender, ethnicity, and place of residence [31]. In this study, we analyzed the risk factors for the development of all cancers, not only NHL. Older age, leukopenia and thrombocytopenia, and use of corticosteroids were related to the development of malignancies.

In contrast to hematologic malignancies, data on solid cancers seen in the subjects with pSS are not consistent. There are incompatible data in the literature regarding solid tumors and their incidence in patients with pSS. Some studies revealed a high incidence of several organ-specific malignancies such as thyroid, breast, lung, oral cavity, and stomach cancers. A recently published study showed an elevated risk for oropharyngeal cancer, as well as lung cancer in males, and thyroid cancer in female patients [27]. Another study revealed an increased risk for the development of thyroid, lip, oral cavity, and stomach cancers [16]. In a metaanalysis, it was reported that only the risk for developing thyroid cancer was increased among solid tumors, and the SIR was found to be 0.61 (95% CI 0–1.21) for ovarian cancer [9]. Brito-Zerón P et al. did not detect an increased risk for ovarian (SIR; 0.46, 95% CI 0.07–3.28) and breast (SIR; 0.89, 95% CI 0.53–1.51) cancers [16]. In this study, the most common solid cancers were breast (n = 6) and ovarian cancers (n = 6), and there was a significant relationship between pSS and the risk of ovarian cancer (SIR 12.76; 95% CI 2.545–22.975).

There is an increased risk for the development of hematological malignancies in autoimmune diseases, which provide a perfect environment for tumor development via loss of self-tolerance and chronic immune dysregulation [32]. The immunopathogenetic pathways of pSS overlap with SLE and two diseases may occur together [33]. SLE is also associated with the overall increased cancer risk -a 4-fold increased risk for NHL-compared with the general population. The potential mechanisms for the development of malignancy are dysfunctions of the immune system, cytokines, and other pathways [34]. The risk of developing hematological cancers in patients with pSS and SLE is higher, and they may develop through common pathogenesis in both disorders. A decreased risk for breast, ovarian, and endometrial cancers was reported in the subjects with SLE [35]. It was claimed that this risk reduction might be related to hormonal factors or lupus-related antibodies [34]. Breast cancer is among the most commonly encountered cancers in patients with systemic sclerosis and inflammatory myopathies [36,37]. Like SLE, the risk of breast cancer is reduced in RA [38]. The risk for ovarian cancer is also increased in patients with inflammatory myopathies [36]. In the light of this information, it can be concluded that, unlike hematological cancers, the development of solid cancers can be associated with different factors and mechanisms underlying the pathogenesis of autoimmune diseases.

Ovarian cancer risk increases with older ages, and it is more prevalent in western countries. It can be related to reproductive period, the number of births, breastfeeding, genetics, and environmental factors such as diet [39]. Chronic inflammation could be associated with an increased risk of ovarian cancer via cyclo-oxidase enzyme, arachidonic acid, and prostaglandins as well as increased estrogen levels. Regular acetylsalicylic acid intake is associated with the reduction in ovarian cancer risk [40]. Regular exercise reduces the risk of cancers, including ovarian cancer [41]. Hormonal contraception in any period of life reduces the risk of developing ovarian cancer [42]. In this study, we did not investigate the causes of solid cancers, including ovarian cancer. In addition to known risk factors, chronic inflammation, inactivity due to joint problems, and avoidance of hormonal contraception in the patients with pSS may contribute to the increased risk of ovarian cancer. Further studies are necessary to determine pSS-related risk factors for ovarian cancer.

There are some limitations of this study; first, the limited number of patients made statistical analysis difficult. Especially, the number of male patients with pSS was low in this cohort. It was impossible to calculate the SIR of cancer types with less than four patients because of the wide range of confidence intervals and the standard error. Secondly, the study was conducted retrospectively, therefore, data on comorbidities known to be associated with cancers such as smoking and drinking habits, physical activity, and disease activity could not be collected and analyzed. Thirdly, since disease activity for pSS could not be calculated for all patients, the relationship between disease severity and cancers could not be examined. Fourthly, due to lack of information about regional cancer incidence in Antalya, where the data were collected, cancer incidence in the general population was obtained from GLOBOCAN 2018 Turkey database. Nevertheless, since this study was conducted in a wide region of Turkey with 2.5 million people, the results may reflect the situation of the Turkish population with pSS.

In conclusion, data on the development of solid and hematological malignancies in our pSS cohort are presented in this article. The prevalence of malignancy in the patients with pSS was found to be 7.9%, and the overall cancer risk was found to increased by 2.45-fold. The risk for the development of ovarian cancer was significantly increased in the subjects with pSS. Further studies are required to reveal pSS-related factors playing a role in the development of ovarian cancer.

## Figures and Tables

**Table 1:** Comparison of the characteristics of pSS patients with and without cancer.

	All patients		pSS patients				
			without cancer		with cancer		p
n	430		396		34		
Age, years, (mean±SD)	58.3±12.0		57.9±12.0		66.2±10.2		**< 0.001**
Duration of disease, (mean±SD)	7.41±4.7		7.25±4.6		9.30±5.2		**0.003**
	n/n	%	n/n	%	n/n	%	
Female	396/430	92.1	366/396	92.4	30/34	88.2	0.385
Dry mouth	413/430	96.0	380/396	69.0	33/34	97.1	0.768
Dry eyes	378/430	87.9	346/396	87.4	32/34	94.1	0.856
Focus score ≥ 1	256/303	85.0	232/276	84.1	24/27	88.8	0.756
ANA (>1/100 dilution) (+)	267/425	62.8	247/391	63.2	20/34	58.8	0.615
Anti-Ro (+)	181/377	48.0	166/346	47.9	15/31	48.4	0.341
Anti-La (+)	51/402	12.7	45/371	12.1	6/31	19.4	0.246
Rheumatoid factor (+)	79/342	23.1	69/308	22.4	10/34	29.4	0.210
Anemia	107/373	28.7	97/340	28.5	10/33	30.3	0.491
Leukopenia	66/425	15.5	57/392	14.5	9/33	27.3	0.052
Lymphocytopenia	61/397	15.4	54/368	14.7	7/29	24.1	0.174
Thrombocytopenia	21/424	5.0	15/391	3.8	6/33	18.2	**<0.001**
Hypergammaglobulinemia	70/289	24.2	65/264	24.6	5/25	20.0	0.775
Low C3/C4	33/357	9.2	28/330	8.5	5/27	18.5	0.084
Arthralgia and/or arthritis	236/373	63.3	217/343	63.3	19/30	63.3	0.994
Raynaud’s phenomenon	44/360	12.2	39/332	11.7	5/28	17.9	0.379
Pulmonary involvement	63/381	16.5	57/349	16.3	6/32	18.8	0.725
Elevated ESR (at the diagnosis)	225/376	59.8	209/351	59.5	16/25	64.0	0.661
Elevated CRP (at the diagnosis)	134/377	35.5	120/352	34.1	14/25	56.0	**0.027**
Use of corticosteroids	106/387	27.4	94/360	26.1	12/27	44.5	**0.039**
Use of hydroxychloroquine	345/375	92.0	32/349	91.7	24/26	92.3	0.952
At least one immunosuppressant*	100/363	27.5	88/336	26.2	12/27	44.4	**0.041**

pSS, primary Sjögren syndrome; n, number of patients; SD, standard deviation; ANA, antinuclear antibodies; C3, complement 3; C4, complement 4; ESR, Erythrocyte sedimentation rate; CRP, C-reactive protein;

* include azatiopürine, methotrexate, rituximab and cyclophosphamide

**Table 2:** The distribution of cancers in pSS patients.

	pSS cohort			
	Overall (n = 430)	Female (n = 396)	Male (n = 34)	p
**All cancers (n, %)**	**34 (7.9%)**	**30 (7.6%)**	**4 (11.8%)**	**0.330**
**Solid cancers (n, %)**	**26 (6.0%)**	**22 (5.6%)**	**4 (11.8%)**	**0.147**
Breast (n)	6	6	-	
Ovarian (n)	6	6	-	
Lung (n)	2	2	-	
Thyroid (n)	2	2	-	
Pancreas (n)	2	-	2	
Stomach (n)	2	1	1	
Skin (n)	1	1	-	
Colorectal (n)	1	-	1	
Corpus uteri (n)	1	1	-	
Bladder (n)	1	1	-	
Brain (n)	1	1	-	
Renal cell (n)	1	1	-	
**Hematological (n, %)**	**8 (1.8%)**	**8 (2.0%)**	**-**	**1.000**
NHL (n)	4	4	-	
Leukemia (n)	3	3	-	
MM/ID (n)	1	1	-	

pSS, primary Sjögren syndrome; NHL, Non-Hodgkin lymphoma; MM/ID, myeloma multiple and malignant immunoproliferative diseases.

**Table 3:** Standardized incidence ratios (SIRs) for cancers in pSS patients.

Cancer Categories	Total (n = 430)				Women (n = 396)				Men (n = 34)			
	Incidence in the general Turkish population	pSS cohort			Incidence in the Turkish women population	pSS cohort			Incidence in the Turkish men population	pSS cohort		
		Obs	Exp*	SIR (95% CI)		Obs	Exp*	SIR (95% CI)		Obs	Exp*	SIR (95% CI)
**All cancers**	**363.2**	**34**	**13.85**	**2.45 (1.625–3.275)**	**292.8**	**30**	**10.31**	**2.91 (1.869–3.951)**	**460.9**	4	1.35	2.96 (0.056–5.864)
**Solid cancers**	**292.3**	**25**	**11.15**	**2.42 (1.542–3.298)**	**254.2**	**21**	**8.95**	**2.35 (1.347–3.353)**	**398.8**	4	1.18	3.39 (0.068–6.712)
Breast	75.9	6	2.89	2.08 (0.419–3.741)	75.9	6	2.67	2.25 (0.452–4.048)	-	-	-	-
Ovarian	12.3	6	0.47	**12.76 (2.545–22.975)**	12.3	6	0.43	**13.95 (2.785–25.115)**	-	-	-	-
**Hematological**	**24.1**	**8**	**0.92**	**8.42 (2.394–14.446)**	**20.2**	**8**	**0.71**	**11.27(3.462–19.078)**	**28.8**	**-**	**0.08**	**-**
NHL	9.6	4	0.37	10.81 (0.216–21.404)	8.2	4	0.29	13.79 (0.276–22.310)	11.2	-	0.03	-

Obs, observed; Exp, expected; SIR, standardized incidence ratio; CI, confidence interval; NHL, Non-Hodgkin lymphoma

* Calculated using data from GLOBOCAN 2018 (Global Cancer Observatory, International Agency for Research on Cancer, World Health Organization Cancer Today. https://gco.iarc.fr/) for +20 years olds.

Obs, observed; Exp, expected; SIR, standardized incidence ratio; CI, confidence interval; NHL, Non-Hodgkin lymphoma

* Calculated using data from GLOBOCAN 2018 (Global Cancer Observatory, International Agency for Research on Cancer, World Health Organization Cancer Today. https://gco.iarc.fr/) for +20 years olds.

**Table 4:** Logistic regression analysis of the risk factors for cancers in pSS patients.

	B	t	95% Convidence Interval		p
Gender	0.050	0.769	−0.094	0.215	0.443
Age	0.191	2.837	0.001	0.008	**0.005**
Disease duratiom	0.139	1.916	0.000	0.019	0.057
Antinuclear antibodies (+)	−0.021	−0.336	−0.055	0.039	0.737
Rheumatoid factor (+)	−0.024	−0.384	−0.025	0.017	0.701
Anti-Ro (+)	−0.015	−0.215	−0.015	0.012	0.830
Anemia	−0.026	−0.391	−0.103	0.069	0.696
Leucopenia	0.143	2.181	0.010	0.205	**0.030**
Thrombocytopenia	0.236	3.596	0.128	0.440	**<0.001**
Low complement levels	0.090	1.348	−0.037	0.198	0.179
Raynaud’s phenomenon	0.000	0.000	−0.113	0.113	1.000
Hypergammaglobulinemia	−0.082	−1.273	−0.119	0.026	0.205
Pulmonary involvement	−0.009	−0.139	−0.106	0.092	0.889
Use of corticosteroids	0.333	4.008	0.002	0.006	**<0.001**
Use of hydroxychloroquine	0.001	0.008	−0.002	0.002	0.993
Use of immunosuppressant	−0.147	−1.896	−0.003	0.000	0.059
